# Towards Precritical Medical Therapy of the Abdominal Aortic Aneurysm

**DOI:** 10.3390/biomedicines10123066

**Published:** 2022-11-29

**Authors:** Lucia Musumeci, Wolf Eilenberg, Joël Pincemail, Koichi Yoshimura, Natzi Sakalihasan

**Affiliations:** 1 Department of Cardiovascular Surgery, CHU Liege, 4000 Liege, Belgium; 2 Department of General Surgery, Division of Vascular Surgery, Medical University of Vienna, 1090 Vienna, Austria; 3 Department of Medical Chemistry, CHU Liege, 4000 Liege, Belgium; 4 Department of Surgery and Clinical Science, Yamaguchi University Graduate School of Medicine, Ube 755-8505, Japan; 5 Graduate School of Health and Welfare, Yamaguchi Prefectural University, Yamaguchi 753-8502, Japan

**Keywords:** abdominal aortic aneurysm, AAA, inflammation, intraluminal thrombus, ILT, antioxidant, polyphenols, metformin, antiplatelet therapy, thromboxane A2, BM-573, microRNAs, antagomir

## Abstract

Pharmacotherapy for abdominal aortic aneurysm (AAA) can be useful for prevention, especially in people at higher risk, for slowing down AAA progression, as well as for post-surgery adjuvant treatment. Our review focuses on novel pharmacotherapy approaches targeted towards slowing down progression of AAA, known also as secondary prevention therapy. Guidelines for AAA are not specific to slow down the expansion rate of an abdominal aortic aneurysm, and therefore no medical therapy is recommended. New ideas are urgently needed to develop a novel medical therapy. We are hopeful that in the future, pharmacologic treatment will play a key role in the prevention and treatment of AAA.

## 1. Introduction

Research conducted over the last 25 years has advanced our understanding of the pathophysiology underlying the progression of abdominal aortic aneurysm (AAA) [1]. Onset of human aortic aneurysms may be due to multifactorial causes, but, in spite of this, most aneurysms share common pathological processes. Different types of inflammatory mediators activate signalling pathways, shifting the balance of extracellular matrix metabolism (ECM) toward degradation, thereby leading to the progression of AAA. Many candidate drugs for treatment of AAA are effective to suppress the inflammatory signalling, while other candidate drugs are targeted towards ECM degradation [2].

Although the advancement in AAA pathophysiology research, there is no pharmacological treatment that would block progression or revert aneurysms. The actual treatment strategy relies on either open surgery or endovascular aneurysm repair (EVAR) for patients whose aneurysm is over or equal to 55 mm for men and 50 mm for women, while for patients whose aneurysms are below the surgical threshold (small to medium-sized AAA: <50 mm) at the moment there is no preventive therapy, but only surveillance using Ultrasounds (US) and/or Computer Tomography (CT), especially when size approaches threshold surgical values [1]. Surveillance follow-up by US is performed every 2–3 years when diameter is <40 mm and every 6–12 months when diameter is between 40 and 45 mm for both for men and women, while every 3 months for women and every 6 months for men whose aortic diameter is between 45 and 50 mm, finally every 3–6 months for men whose diameter is between 50 and 55 mm [1].

We are hopeful that in the future, pharmacologic treatment will play a key role in the primary, secondary, and tertiary prevention of AAA [3].

In primary prevention, the goal of pharmacotherapy is to reduce the incidence of AAA. Although the primary cause of AAA onset remains unclear, several known risk factors contribute to AAA development, such as hypertension and hypercholesterolemia. The incidence of AAA might be reduced by appropriate pharmacologic treatment of these risk factors.

The goal of pharmacotherapy for the secondary prevention of AAA is to reduce disease progression and the risk of surgical referral in patients with small AAA. Pharmacologic therapy to prevent AAA progression, or ideally to reverse AAA formation, would have a huge impact on the management of patients with small AAA once it is established, and become a first-line treatment, replacing surveillance and early repair [4].

In tertiary prevention, the goal of pharmacotherapy is to reduce AAA-related complications and mortality in patients with large AAA. Pharmacotherapy for tertiary prevention is expected to serve as an adjuvant treatment to reduce peri- and post-operative complications, as well as to improve the results of EVAR. Because AAA patients often have several comorbidities that significantly affect the outcome of AAA repair, preoperative care strategies, especially pharmacotherapy, may help improve post-intervention morbidity and mortality. In addition, ongoing aortic wall degeneration and the subsequent failure of aneurysm exclusion, such as endoleak, is a major concern after EVAR. To address this concern, adjuvant pharmacotherapy concomitant to or after EVAR could provide an ideal solution [5].

## 2. Focus on Pharmacologic Therapies in Secondary Prevention

A key limitation of contemporary treatment strategies of AAA is the lack of therapy directed at small AAAs to slow down progression and thereby prevent the need for later major surgical repair or life-threatening rupture.

In fact, several challenges need to be addressed [3]. First, it is possible that appropriate drug targets in human AAA have not been identified. Indeed, many animal studies have assessed the effects of interventions in limiting AAA development rather than the effects on pre-established AAA [6]. Continued efforts are essential, including the appropriate use of animal models and human samples. Second, few pharmacokinetic approaches have been tested [6]. Since AAA is predominately localized to a limited site on the aorta, local drug delivery is reasonable in order to increase therapeutic efficacy and reduce systemic side effects. Third, there is a possibility that the heterogeneity of human AAA might not be taken into account when testing therapeutic agents. Therefore, identifying biomarkers that accurately reflect biological activity in AAA would allow the development of tailor-made AAA treatments.

## 3. Latest Guidelines

According to the 2014 European Society of Cardiology (ESC) Guidelines, smoking cessation is strongly recommended to slow AAA growth, while statins and angiotensin-converting enzyme (ACE) inhibitors use, to reduce the risk of aortic complications in patients with small AAA, is proposed only as a very weak recommendation [7]. Smoking cessation is strongly recommended also in the 2018 Society for Vascular Surgery (SVS) Guidelines to reduce the risk, not only of AAA growth, but also rupture. There is also a strong recommendation for beta blocker therapy not to be used for the sole purpose of reducing the risk of AAA expansion and rupture. On the other hand, statins and ACE inhibitors should be considered because of the potential benefits to cardiovascular disease, but these drugs should not to be used for the sole purpose of reducing the risk of AAA expansion and rupture (weak level of recommendation) [8]. According to the European Society for Vascular Surgery (ESVS) 2019 Clinical Practice Guidelines, smoking cessation is also strongly recommended to reduce the rate of aneurysm growth, as well as risk of rupture, and patients should receive help to achieve this. No specific medical therapy has been proven to slow the expansion rate of an abdominal aortic aneurysm, and therefore no medical therapy is recommended. Strategies targeted at a healthy lifestyle, including exercise and a healthy diet, should be considered to reduce cardiovascular risk in patients with abdominal aortic aneurysm. Likewise, blood pressure control, statins and antiplatelet therapy are also recommended because of the potential benefits to cardiovascular disease [9].

Taken together, the present state of the art treatment of AAA is limited to best medical care for comorbidities, life style changes and ultimately surgery, once the threshold in aneurysm size is reached. Despite initial enthusiasm, different classes of existing cardiovascular medication including: statins, platelet aggregation inhibitors, beta blockers and ACE inhibitors may improve survival regarding overall cardiovascular risk, but they have not shown any significant impact on AAA disease progression in randomised trials.

New ideas are urgently needed to develop a novel medical therapy for this important disease to replace the current “watchful waiting” approach.

## 4. Novel Therapeutic Options in AAA

### 4.1. Dietary Polyphenols

Human studies confirmed that pathological oxidative stress plays a pivotal role in AAA pathogenesis [10]. Pathological oxidative stress (OS) is defined as an imbalance between reactive oxygen species (ROS) and antioxidants, in favour of the former, leading to a disruption of redox signalling with consequence of oxidative damage to lipids, proteins and DNA. Increased activity of NAD(P)H oxidase, inflammation, over-expression of inducible nitric oxide synthase (iNOS), iron release from hemoglobin and endothelial dysfunction resulting in uncoupled endothelial nitric oxide synthase (eNOS) are recognized to be important sources of ROS production in AAA [10,11]. Therefore, reducing OS using antioxidants and especially dietary polyphenols could represent a potential strategy for limiting AAA development. Epidemiological studies performed by the group of Kent et al. in a cohort of more than 3 million individuals [12] and in the prospective Cohort of Swedish Men and the Swedish Mammography Cohort (*n* = 44,317 women and 36,109 men aged 46–84 years followed for thirteen years) [13] have shown that consumption of 5 servings of fruits and vegetables per day and moderate red wine containing anthocyanins [14] was associated with reduced risk of developing AAA. In a recent study on 1781 AAA cases [15], it has been shown that the adherence to a Mediterranean diet rich in polyphenols present in fruits and vegetable, spices, olive oil and moderate red wine was inversely associated with AAA incidence in current and ex-smokers. In this last cohort, it has also been shown that tea consumption, rich in catechins, up to ≥2 servings per day, was associated with a lower risk of both non-ruptured and ruptured AAA [16]. From a mechanistic point of view, diet polyphenols may potentially interfere through their antioxidant properties with many factors involved in AAA development by: reducing inflammation [17]; restoring endothelial function, [18] which is known to be altered in AAA [19]; decreasing DNA global methylation [20,21]; and protecting against telomere attrition [22,23].

Moreover, an important property of polyphenols is to stimulate the Keap1/Nrf2/ARE pathway [24]. By autooxidation in the cells polyphenols generate moderate production of ROS which leads to the separation of Keap 1 and NrF2. This last one acting as transcription factor migrates into the nucleus where it binds to DNA. This results in the overexpression of genes stimulating a large number of antioxidant enzymes having a higher capacity to eliminate ROS than low molecular weight antioxidants such as vitamins C and E. Of interest is to note that the lack of Nrf2 transcriptional activity is associated with AAA formation [25].

Two surgical methods of repair are available for AAA, open aneurysm repair and endovascular aneurysm repair (EVAR). Increased oxidative stress in response to the ischaemia has been evidenced in both procedures, but to lesser extent in EVAR [26]. Concomitantly, a depletion in antioxidants was observed [27]. Several studies have evidenced that supplementation with different antioxidants (vitamin E, ubiquinone) was able to reduce the oxidative stress during AAA repair [28].

In conclusion, a diet enriched in polyphenols should not be neglected in the context of the prevention of aneurysms and could represent an adjuvant therapy. However, further studies are required to evaluate more precisely the potential impact of the pharmacological use of enriched polyphenol vegetable extracts on AAA prevention and progression. In case of surgical procedure for AAA repair, it should be recommended to pre-treat the patient with a supplementation of low molecular weight antioxidants such as vitamins C and E or ubiquinone.

### 4.2. Metformin

Metformin has been proposed as a potential new treatment option for small AAAs. Metformin could be the first conservative treatment option for AAA that targets (among other pathways) the driver of the disease-chronic inflammation. Based on the observed “off-target” effect of metformin on aneurysm growth in diabetic AAA patients, this drug repurposing effort meets major, presently unsolved problems of AAA: it can be expected to reduce the progression of the disease, which in turn may limit patient need for surgical repair and reduce the risk of vessel rupture. Metformin is a drug, that is readily supplied by almost thirty pharmaceutical companies such as Merck, TAD Pharma and Sandoz. It is inexpensive and has a well-established safety profile with few side effects during long-term therapy. The most common side effects of metformin include digestive problems (diarrhoea, nausea and flatulence) as well as vitamin B-12 deficiency. While gastrointestinal complications are expected to occur in about 10% of individuals and may be controlled by dose titration, vitamin B-12 deficiency affects 5% of metformin-treated patients and is compensated by vitamin supplementation [29]. This drug is well established as an anti-diabetic medication and is clinically safe. In 4 retrospective observational studies [30,31,32,33], metformin was associated with significantly reduced aneurysm progression in diabetic AAA patients as well as reduced incidence of aneurysm rupture and surgical repair (summarized in Table 1). It should be noted that an additional Danish study on diabetic patients reported a protective effect of metformin against AAA rupture with an odds ratio of 0.74 which was, however, not statistically significant [34]. Furthermore, a pre-clinical experimental AAA model demonstrated the therapeutic efficacy of metformin to prevent aneurysm formation in normoglycaemic mice with elastase induced AAA [33]. Thus, based on the clinical evidence with diabetic AAA patients and the preclinical results from normoglycemic mouse models, metformin seems to have a high potential to limit AAA growth in non-diabetic AAA patients. It is important to note that existing literature [9] supports the notion that a significant impact on AAA diameter will translate into improved clinical end points (rupture and indication for open or endovascular surgical repair). Metformin is the most commonly prescribed anti-diabetic medication worldwide; it is a well-established and well-tolerated drug which has been applied in the clinic for over 60 years [35]. This compound acts via multiple pathways. It is known to decrease mitochondrial oxidative phosphorylation, thereby lowering cellular ATP-to-AMP levels and driving AMPK activation which blocks liver gluconeogenesis and lipogenesis. Furthermore, metformin inhibits inflammation, reduces angiogenesis and supports autophagy and tissue regeneration [36]. Thus, metformin treatment has also been tested for diseases other than diabetes. For example, therapeutic efficacy has been shown in a phase II clinical study in systemic lupus erythematosus (SLE). Metformin significantly decreased clinical flares as well as requirement for co-medication, i.e., prednisolone exposure for SLE patients [37]. Comparable to SLE, AAA is a disease with a chronic inflammatory component and hence, the vasculo-protective functions attributed to metformin (involving inflammation, tissue regeneration and energy metabolism) may also prove beneficial in this setting. Summarising, 4 retrospective analyses on metformin prescription to diabetic AAA patients (Table 1) revealed a significant reduction of AAA progression, rupture rate or incidence of repair when compared to AAA patients treated with other anti-diabetic drugs [31,32,33]. While the metformin effect ranged from 20–76% decrease in annual aneurysm growth (which may in part relate to patient selection, sample size or imaging method), metformin consistently displayed a protective effect. Furthermore, the experimental model of AAA development in normoglycaemic mice provided pre-clinical evidence that metformin therapy might also be beneficial for AAA disease control in the absence of diabetes [33]. A small-scale national Austrian trial, Vienna-MetAAA-trial [38] has been initiated in Vienna, Austria, to collect preliminary compliance and tolerability data which have proven highly satisfactory. The results of the Vienna-MetAAA-trial are expected to be published beginning of 2023. Next to Vienna, Standford University, one of USA leading vascular surgeons Prof. Ronald Dalman is recruiting for a large randomized controlled trial [39]. In Europe, Uppsala, Sweden, Jon Unosson, Anders Wanhainen, lead author in the ESVES guidelines, conduct the large MAAAGI-trial [40] an open labelled metformin observance study.

Among antidiabetic drugs, good candidate drugs for AAA treatment option are also flozins, a class of sodium glucose transporter protein-2 (SGLT-2) inhibitors reported to reduce the risk of cardiovascular diseases [41]. In a recent preclinical study, dapagliflozin resulted effective in limiting initiation and progression of AAA in an elastase normoglycaemic mouse model, probably via a mechanism involving inflammation and angiogenesis [42].

### 4.3. Antiplatelet Therapy: Targeting the TxA_2_ Pathway to Reduce AAA Progression

AAA is an atherothrombotic and inflammatory disease characterized by an irreversible aortic wall remodelling and by the presence of: (1) a non-occlusive intraluminal thrombus (ILT), in most patients, which participates to the progression of the aneurysm; (2) a degraded media, with loss of elastin and collagen, as well as loss of vascular smooth muscle cells (VSMCs); (3) a thicker than normal inflammatory and/or fibrotic adventitia [1,43].

The role of ILT in the aneurysmal process has been shown in several in vivo studies [44], many of which showed that preventing ILT formation, using anti-thrombotic/anti-platelet therapies, protected animals from aneurysms [45]. In rats models the anti-integrin GPIIb/IIIa antibody Abciximab (Reopro) limited ILT formation and prevented aneurysm dilatation [46] and the P2Y12 receptor antagonist Ticagrelor proved protective, as rats developed smaller aneurysms [47].

Platelet activation can also be dampened effectively by targeting enzymes and/or receptors involved in thromboxane A2 (TxA_2_) signalling [48]. TxA_2_ biosynthesis involves mainly cyclooxygenases (COX1 and COX2) catalysing the conversion of arachidonic acid to prostaglandin G2 (PGG_2_) and further to prostaglandin H2 (PGH_2_) and thromboxane synthase (TS), catalysing the conversion of PGH_2_ to TxA_2_.

In animal models of AAA COX inhibitors are efficient in inhibiting aneurysmal growth [49]. In clinical practice, COX2 inhibitors, mainly aspirin, are recommended in patients with aneurysm as a risk reduction strategy, together with statin and antihypertensive therapy, as stated in the latest guidelines on AAA management [9].

Molecules that are at the same time antagonists of TxA_2_ receptor (TP receptor) and inhibitors of TS can have a more pronounced antiplatelet activity, since TP ligands, other than TxA_2_, could still favour a proinflammatory and proatherogenic vascular phenotype. Moreover, TxA_2_ biosynthesis suppression could lead to accumulation of prostaglandin endoperoxide, converted to either PGD_2_ by platelets or PGI_2_ by vessel wall, both of which increase platelet cyclic AMP levels and inhibit platelet activation.

BM-573 is a dual TxA_2_ inhibitor (TP antagonist and TS inhibitor), which was tested and showed to be beneficial in a murine and pig model of myocardial infarction [50,51]. BM-573 was also effective in an animal model of acute pulmonary embolism, where its infusion reduced pulmonary vasoconstriction [52]. BM-573 has been also shown to have an antiplatelet effect in rats when injected intraperitoneally, without affecting bleeding time, and to have a positive effect in a model of thrombosis induced by ferric chloride when applied to the abdominal rat aorta [53]. This molecule bears also antiangiogenic properties, mainly by inhibiting endothelial cell migration [54].

Combination of TxA_2_ blockade with COX-1 inhibition was found to be a more effective therapeutic approach to modulate atherogenesis than suppression of COX-1 activation alone [55].

In another study the effect of BM-573 and Acetylsalicylic acid (ASA) (COX-1 and COX-2 inhibitor) where used alone or in combination on atherosclerotic lesion formation in apolipoprotein E-deficient mice [56].

Considering the high efficacy of BM-573 in mouse models of atherosclerosis and various models of thrombosis, we postulated the hypothesis that this molecule could reduce the development of AAA during the early stage.

We were the first research group to show that in a AAA rat elastase model oral administration of BM-573 (180 mg/kg/day) significantly reduced the development of AAA in more than half of the treated rats and the mean thickness of their ILT was significantly decreased. We also observed a significant reduction in the plasma concentration of thromboxane B2, a metabolite of TxA_2_. The analysis of the aortic wall revealed a downregulation of several inflammatory mediators, like COX2, IL6 and RANTES (Regulated on Activation Normal T cell Expressed and Secreted), a pro-inflammatory cytokine expressed by platelets [50].

A limitation of this treatment is that not all the rats treated with the molecule showed absence of thrombus. In fact, there are other mediators of platelets activation, e.g., ADP and serotonin, released potentially by thrombin-activated platelets, which amplify platelet clot formation independently of TxA_2_.

The usage of drugs, like COX-1 specific inhibitors or P2Y12 inhibitors, in combination with BM-573 would eventually lead to an additive inhibitory effect on the aneurysmal growth.

The challenge in using antiplatelets is that such molecules increase bleeding risk, therefore, a more local action of such inhibitors would be preferred. One can imagine that a patient with a small aneurysm could undergo an endovascular procedure to deliver a locally acting molecule to avoid or at least decrease off-target systemic effects.

### 4.4. MicroRNA Targeting

MicroRNAs (miRNAs) play important roles in the process of AAA formation [57]. Clinical studies and in vivo studies have shown an altered expression of specific miRNAs in circulation and/or aortic aneurysmal wall [58].

MiRNAs are small non-coding double stranded RNA composed of 19–25 nucleotides, they are negative regulators of gene expression at the post-transcriptional level [59], guiding the RNA-induced silencing complex (RISC) to bind the 3′ untranslated regions (UTR) of target mRNA, promoting degradation or inhibiting translation [60]. One miRNA can bind to multiple mRNAs and one mRNA can bind to different miRNAs. They have been discovered in the circulation and have since then been studied as potential candidate biomarkers for several diseases, including AAA disease. MiRNAs are also promising therapeutic targets, since their modulation can affect not only one specific target, but entire functional networks, which, on the other hand, could be a limitation due to increased chance of off-target effects [61].

To identify therapeutic miRNA targets it is important to distinguish AAA causative miRNAs (miRNAs involved in AAA onset) and miRNAs that are dysregulated as a result of the pathology (released by the aneurysmal tissue). Since aging, smoking and being male are the main risk factors, miRNA dysregulated in these conditions could be involved in AAA onset, therefore, modulating their expression could be used as therapeutic strategy for AAA progression.

There are essentially two strategies to counteract the action of miRNA and/or to modulate their levels [61]:

1. miRNA mimics able to increase the levels of the target miRNA. These can be double strand oligodeoxynucleotides (ODN), consisting of a guide strand identical to the endogenous mature miRNA and a passenger strand. They are called pre-miRNA/miRNA-mimics and they act similarly to the mature endogenous miRNA, replacing it [62].
2. miRNA inhibitors able to decrease the levels of the target miRNA. These can be single-strand RNAs with sequence complementary to the target miRNA, which are miRNA antagonist preventing the interaction between the miRNA and their target mRNA, antisense oligonucleotides (ASO) (named also miRNA inhibitor or antagomir) or artificial circular RNA (circRNA) sponges (circmiRs) [63]. Another way to reduce miRNA levels is to use single-strand RNAs complementary to the mRNA, therefore binding to the miRNA binding site (miRNA competitors or block-mir) [63].

Both strategies can be viewed as gene therapy, but to the contrary to gene therapy, where only one gene is targeted, RNA-based therapy can modulate sets of genes. This is useful especially in complex diseases like AAA. Although such advantage, their use in clinic remains challenging due to their easy degradation by endonucleases and to their non-specific effects. To overcome these challenges, two solutions have been proposed: a chemical modification of the ODN that would increase their in vivo stability (locked nucleic acid or morpholino oligomers); a delivery method, like a nanocarrier such as nanoparticles, liposomes and micelles that would protect the nucleic acid from degradation and avoid off-target effects by acting directly on the target tissue/molecule. Such localized effect of the drug can be reached using a tissue-specific delivery system [64,65].

In the context of AAA one of the first miRNA to be described as associated with AAA was miR-29b, whose downregulation was observed in in vivo models [66] and resulted in increased expression of several genes involved in AAA pathophysiology, (*Col1a1*, *Col3a1*, *Col5a1* and *Eln*). Maegdefessel L. et al., [66] found that by further inhibiting the expression of miR-29b, using locked nucleic acid anti-miR-29b, collagen expression increased, leading to a fibrotic phenotype and a reduction of AAA progression, while its overexpression caused expansion of the aorta and rupture. This suggests that the downregulation of miR29b in vivo is a physiologic protective response to avoid expansion and rupture of the aortic wall. Moreover, the same group showed in two murine AAA models and in human aneurysmal tissue an upregulation of miR-21 [67]. Lentivirus-mediated overexpression of miRNA-21 reduced the incidence of AAA and inhibited the expansion of AAA in vivo, suggesting that upregulation of miR-21 is a physiological response to the pathology. On the other hand, they found miR-24 to be inversely correlated with AAA size and its modulation through antagomir or pre-miR proved its downregulation to be pathologic and not physiologic as for miR-21 and miR-29b [58].

Our group [68] looked at circulating miRNA among AAA patients with strong [18F] fluorodeoxyglucose (FDG) uptake, detected by positron emission tomography (PET), compared to AAA patients with low uptake. Since FDG-PET uptake is correlated with aortic wall instability, we wanted to identify specifically miRNAs responsible for the expansion of AAA. We have found 3 downregulated miRNAs, miR-99b-5p, miR-125b-5p, and miR-204-5p, which were also significantly reduced in the aneurysmal tissue and were inversely correlated with the expression of some of their potential gene targets, most notably matrix metalloproteinase 13 (MMP13). Moreover, we found two miRNAs to be upregulated (miR-33a-5p and miR-142-5p). In support of our findings, another study reported miR-33a-5p as upregulated in patients with AAA compared to healthy controls [69]. MiR-33 has been reported to have a role in cholesterol homeostasis [70] and its genetic ablation in angiotensin II– and calcium chloride–induced AAA mouse models attenuated inflammation and AAA formation [71]. This suggests that upregulation of miR-33 is pathogenic and could be targeted using antagomirs and eventually this strategy would work also in humans using an antagomir against miR-33a-5p.

To our knowledge there are no clinical trials using antagomir or ODN to treat AAA, even though clinical trials using ASO and ODN are reported for other diseases and there are still not enough studies of miRNAs in AAA, probably because of the challenges in validating their significance into the disease onset and progression [72].

## 5. Conclusions

The AAA is a serious medical problem that affects a significant proportion (around 4% prevalence) of men with more than 60 years old. Repair before rupture is rather safe but represents a heavy burden for the health care system. Repair upon rupture is ±4 times more expensive and is associated with high mortality and morbidity [1]. Much can be done to reduce these preventable deaths and the cost to society by devising more efficient procedures for both screening and monitoring the disease and by replacing surgery by pharmacological therapy. Surgery in peptic ulcers has been replaced by pharmacological therapy in recent decades. By stopping and/or retarding AAA growth our aim would be to replace open or endovascular surgical repair of AAA by less invasive, more cost-effective therapies.

## Figures and Tables

**Table 1:** Summary of retrospective analyses on aneurysm progression and AAA related clinical events comparing diabetic AAA patients with and without metformin therapy (95% CI, 95% confidence interval; HR, hazard ratio; SD, standard deviation; SE, standard error).

AAA Study	Diabetic Patients (+/− Metformin)	Primary Outcome Parameter	Effect of Metformin	Reduction (%)
Golledge J. et al. [30]	234	AAA repair or AAA mortality:		
	(129/105)	adjusted HR (95% CI)	0.63 (0.44–0.93)	37%
		compared to non-diabetic AAA		
Itoga N.K. et al. [31]	13,834	Growth of maximal aortic	1.2 ± 1.9 vs.	20%
	(5492/8342)	diameter: mean ± SD	1.5 ± 2.2 mm/y	
Golledge J. et al. [32]	Cohort #2	Growth of maximal aortic	1.40 ± 2.99	
	69	diameter:	vs.	36%
	(39/30)	mean ± SD	2.18 ± 2.96	
			mm/y	
Fujimura N. et al. [33]	58	Growth of maximal aortic	0.4 ± 0.6 vs.	76%
	(15/43)	diameter: mean ± SE	1.7 ± 0.5 mm/y	
